# Eco-Efficient Biosorbent Based on *Leucaena leucocephala* Residues for the Simultaneous Removal of Pb(II) and Cd(II) Ions from Water System: Sorption and Mechanism

**DOI:** 10.1155/2019/2814047

**Published:** 2019-01-02

**Authors:** C. A. Cimá-Mukul, Youness Abdellaoui, Mohamed Abatal, Joel Vargas, Arlette A. Santiago, Jesús Alberto Barrón-Zambrano

**Affiliations:** ^1^Facultad de Ingeniería Química, Universidad Autónoma de Yucatán, Periférico Norte, Kilómetro 33.5, Chuburná de Hidalgo Inn, Mérida, Yucatán 97203, Mexico; ^2^Facultad de Ingeniería, Universidad Autónoma de Yucatán, Av. Industrias no Contaminantes por Periférico Norte Apartado Postal 150 Cordemex, 97310 Mérida, Yucatán, Mexico; ^3^Facultad de Ingeniería, Universidad Autónoma del Carmen, 24155 Ciudad del Carmen, Campeche, Mexico; ^4^Instituto de Investigaciones en Materiales, Unidad Morelia, Universidad Nacional Autónoma de México, Antigua Carretera a Pátzcuaro No. 8701, Col. Ex Hacienda de San José de la Huerta, 58190 Morelia, Michoacán, Mexico; ^5^Escuela Nacional de Estudios Superiores, Unidad Morelia, Universidad Nacional Autónoma de México, Antigua Carretera a Pátzcuaro No. 8701, Col. Ex Hacienda de San José de la Huerta, 58190 Morelia, Michoacán, Mexico

## Abstract

*Leucaena leucocephala* is a potential source of polyphenols widely available in southern Mexico. This work highlights the extraction of polyphenols from *Leucaena leucocephala* leaves waste (LLEPs) and the evaluation of their efficiency to remove the single and multicomponent Pb(II) and Cd(II) metal ions from aqueous solutions. Batch test conditions were carried out to examine the effects of contact time, initial metal ion concentration, and adsorbent dosage on the biosorption process. The surface textures and the composition of the LLEP biosorbent was characterized using pH of point of zero charge (pH_PZC_), attenuated total reflectance Fourier transform infrared (ATR-FTIR), and matrix-assisted laser desorption/ionization time of flight (MALDI-TOF) mass spectrometry, respectively. Further analysis using ATR-FTIR after adsorption contact of biosorbent was also investigated. The highest Langmuir saturation monolayer adsorption capacity, *q*_m_, for the removal of Pb(II) by LLEPs was obtained as 25.51 and 21.55 mg/g in mono- and bimetal solutions, respectively. The pseudo-second-order model provided the best fit for the kinetic data obtained for the removal of Pb(II), Cd(II), and their mixture, and the k_2_ values depend on the adsorbent mass. This implied that the chemisorption process might be the mechanism of the solute ions-LLEPs interaction in this study. Furthermore, nearly 100% removal of lead and cadmium individually and 95% of their mixture was found using 0.9 g of LLEPs.

## 1. Introduction

Biosorption has emerged as a potential and promising solution to remove toxic heavy metals from water and wastewater, and is also considered as an alternative process to the conventional methods such as those based on ion exchange, precipitation, membranes, and electrochemistry processes widely applied in industrial effluents treatments which are very costly and have many limitations [1, 2]. Recent research studies have directed attention to biosorption processes owing to the high metal-binding capacities of various biosorbent materials [3]. Algae-, fungi-, agricultural-, and forestry-based materials have proved to be eco-efficient and environmentally friendly sorbent for heavy metals [3, 4]. Diverse waste materials have been used as low-cost bioadsorbents for heavy metals from water solutions; in particular for lead Pb(II) and cadmium Cd(II) ions, they have used fruit shell [5], algae, eggshell, rice husk, sawdust [6], corn cob [7], olive oil by-products [8], livestock waste [9], forest by-products and waste [10–14].

The use of polyphenols as biosorbents has been widely studied in water and wastewater treatment to remove heavy metals due to the large availability of these compounds in plant material that can be easily extracted from agricultural waste using a green solvent. Several studies have been carried out on the extraction of polyphenols from various plants using different solvents, showing that the structure, yield, recovery, and type of phenolic compounds depend on the plant nature and extraction solvents [15]. *Nepeta melissifolia*, *Phlomis lanata*, *Ilex paraguariensis*, and *Origanum vulgare* demonstrated a high total phenol content with more than 15.0 mg gallic acid equivalent (GAE)/g dried sample, whereas *Geranium purpureum*, *Matricaria chamomilla*, and *Lavandula vera* showed a content less than 7 mg GAE/g dried sample [16, 17].

*Leucaena leucocephala* as a potential source of polyphenols is a fast-growing, tropical, leguminous tree species that belongs to *Mimosaceae*, native to southern Mexico (Yucatán Peninsula), and then extends to Nicaragua, including Guatemala, Honduras, El Salvador, and northern Central America [18, 19]. *Leucaena leucocephala*, named the miracle tree, is considered as a multipurpose tree species for its wider usages as timber and firewood, in pulp and paper industry [20] also as a biomass and protein source for animal feed with yield production of 50 ton ha^−1^ year^−1^ under Mediterranean conditions [21]. Their leaves and seeds are composed mainly of lipids, crude protein, carbohydrates, mimosine, saponins, coumarins, flavonoids, cardiac glycosides, steroids, and phenols and have a high condensed tannin content [18, 22, 23]. Recently, Abu Zarin et al. reported the total phenolic content in *Leucaena leucocephala* hybrid-Rendang from its crude extract to be 3.21 mg GAE/g [22].

Polyphenols, extracted from plants, are characterized by the abundant phenolic hydroxyls that are capable of chelating with transition-metal ions, especially for those metal species with *d*-orbits [24]. Because of the strong chelating ability of phenolic hydroxyls towards transition metals, the polyphenol-based adsorbent showed to be potential and efficient sorbent for the removal of Pb(II) and Cd(II) from aqueous solutions. In this context, Copello et al. studied the application of hybrid low-cost SiO_2_/polyphenol matrices as a bioadsorbent for Pb(II), Cr(III), and Cr(VI) [16]. Waseem et al. have used biomass derived from of *Acacia nilotica* leaves as an adsorbent material for the removal of cadmium and lead, and they found the maximum adsorption to be 2.51 mg/g and 4.99 mg/g for Pb(II) and Cd(II), respectively [13]. Recently, Zhang el al. have evaluated the effectiveness of *Sagittaria trifolia L.* stalk plant to remove Pb(II), Cd(II), and Cr(III) [14]. However, the maximum adsorption capacities of Pb(II) reported by Jayaram and Prasad were 45.454 mg/g using *Prosopis juliflora* as a source of polyphenols [25].

However, to the best of our knowledge, there are no reports of polyphenols extracted from *L*. *leucocephala* as potential metal removers from mono- and multicomponent systems. Indeed, *L*. *leucocephala* residues have no application for human consumption here in Mexico; therefore, it is expected that it will remain a waste that could be considered a sustainable low-cost source of polyphenol adsorbents. Therefore, the aim of this work was to describe the simultaneous sorption mechanism of Cd and Pb from aqueous solution using *L. leucocephala*-extracted polyphenols (LLEPs) considering various experimental parameters such as contact time, adsorbent dosage, and initial concentration.

## 2. Materials and Methods

### 2.1. Chemicals

Deionized water (18.2 M*Ω*/cm^−1^, conductivity) was used for all dilutions and to prepare the synthetic stock solutions of Pb(II) and Cd(II) using PbCl_2_ (98%, Aldrich) and CdCl_2_.2.5H_2_O (79.9 %, J.T. Baker) salts, respectively. Methanol (70%), Folin–Ciocalteu reagent, and Na_2_CO_3_ were used in the procedures for extraction and quantification of total polyphenols.

### 2.2. Plant Preparation

Leaf residues of *Leucaena leucocephala* tree were collected from the campus area of the Autonomous University of Ciudad Del Carmen identified by our botanist Dr. Enrique Lopez, washed with deionized water, dried at 70°C, and then stored in glass bottles to be used for the polyphenols' extraction.

### 2.3. Extraction of Polyphenols

The extraction method of polyphenols was described by Scalbert et al.; 15 g of plant leaf samples was extracted three times with 300 mL of 80% methanol solution under magnetic agitation at room temperature (3 × 2 h). The supernatant was collected after each extraction by filtration and then the sample solution was used for polyphenol quantification. The methanol was evaporated under reduced pressure, and then the polyphenols were dried and ready to be used in the sorption process [26].

### 2.4. Total Polyphenolic Content

Total polyphenolic contents were determined according to the method of Scalbert et al. [26]. 0.5 mL of a 200-fold diluted aqueous extract was mixed with 2.5 mL of 10-fold diluted *Folin–Ciocalteu* phenol reagent and incubated for 1 min, before 2 mL of 7.5% Na_2_CO_3_ was added. The mixture was allowed to stand for 15 min at 50 °C in a water bath and then transferred into cold water. Samples absorbance was measured at 760 nm in a UV-Vis spectrophotometer (Evolution 220) after 30 min of incubation. A gallic acid aqueous solution (80 *µ*g/mL) was used for calibration, and the final results were expressed as mg gallic acid equivalent (GAE) per g of dry weight (DW).

### 2.5. Characterization Methods

#### 2.5.1. Point of Zero Charge (pH_PZC_)

The pH of the point of zero charge (pH_PZC_) of LLEPs has been described as following: 0.10 g of each adsorbent with 50 mL of 0.01 M NaCl adjusted to different initial pH values (pH = 2, 4, 5, 6, 8, 10, and 12). The suspensions were allowed to equilibrate for 24 h under agitation, decanted, and the final pH values of each remaining solution were measured using the pH meter Thermo Scientific (ORION 3-Star pH Benchtop). The experiments were conducted in duplicate, and the measurement temperature was set at 25.0 ± 0.1°C. The pH_PZC_ of the biosorbent was determined from the point of intersection of the curves obtained in the plot of pH_initial_ vs. pH_final_.

#### 2.5.2. MALDI-TOF Mass Spectroscopy

Matrix-assisted laser desorption/ionization time of flight (MALDI-TOF) mass spectrometry was performed on a Bruker Microflex MALDI-TOF mass spectrometer (Bruker Daltonik, Germany) equipped with a nitrogen laser that operates at *λ* = 337 nm. The irradiation target was prepared from a THF solution using 2, 5-dihydroxy benzoic acid (DHB) as matrix. The sample and matrix were combined at a ratio of 2 : 5 *v*/*v*. The measurement was carried out in the linear mode with an acceleration voltage of 20 kV to yield the resulting mass spectrum with a pretty good reproducibility regarding both the ion peaks and their positions.

#### 2.5.3. ATR-FTIR Spectroscopy

Attenuated total reflectance Fourier transform infrared (ATR-FTIR) spectra were collected using a Thermo Scientific Nicolet iS10 FTIR spectrometer fitted with a Thermo Scientific Smart iTR™ ATR accessory with a diamond crystal. Data were collected by an attached computer running OMNIC software. Liquid samples were added directly onto the crystal for analysis at room temperature without applying pressure. Thirty-two spectra were obtained and coadded for each sample covering a range of 4000–650 cm^−1^ at a spectral resolution of 4 cm^−1^. A background spectrum was obtained by collecting 32 coadded scans following cleaning of the diamond crystal with acetone.

### 2.6. Sorption Process

#### 2.6.1. Kinetic

For the sorption kinetics of Pb(II) and Cd(II) by LLEPs, batch mode experiment was performed. 0.1 g of LLEPs was added to 10 mL of Pb(II) or Cd(II) solutions at 100 mg/L. The mixtures were placed in centrifuge tubes and shaken in a rotary shaker for different time intervals (30 min to 24 h). After each specific contact time, the tubes were centrifuged at 3500 rpm for 10 min. The concentration of lead and cadmium metals was determined using a flame atomic absorption spectroscopy (AAS, Thermo Scientific iCE 3000 Series). In order to ensure the truthfulness of experiment results, all experiments were duplicated. The amount of metal ions adsorbed per unit mass of the sorbent was evaluated by using the following equation:

(1) $$\begin{array}{c} q_{\text{t}}=\frac{\left(C_{0}-C_{\text{t}}\right)}{m}V, \end{array}$$

where *C*_0_ (mg L^−1^) and *C*_t_ (mg L^−1^) are the initial and final metal concentration in solution, respectively, *V* is the volume of aqueous phase (L), and *m* is the mass of LLEPs (g).

#### 2.6.2. Isotherms

For isotherms, 0.1 g of LLEPs was mixed with 10 mL of aqueous solution of Pb(II) and Cd(II) with known initial concentrations (10–500 mg L^−1^). The mixtures were shaken for 24 h in a rotary shaker at room temperature. At the end of equilibrium time, the suspensions were centrifuged at 3500 rpm for 10 min, and the concentration of lead metals was analyzed by AAS.

#### 2.6.3. Influence of LLEPs Dosage

In order to study the effect of LLEPs dosage on the Pb(II) and Cd(II) removal efficiency, 10 mL of Pb(II)and Cd(II) solutions with an initial concentration of 100 mg/L was mixed in centrifuge tubes containing 0.1 g, 0.5 g, and 0.9 g of LLEPs. The mixtures were shaken for 30, 60, 180, and 360 min. At the end of each specific contact time, the mixtures were centrifuged, and the metallic ions were determined by AAS.

## 3. Results and Discussions

### 3.1. Total Phenol Content

In order to determine the total phenolic content, the curves of calibration were obtained using known quantities of commercial gallic acid. Total phenolic content of the *L*. *leucocephala* extracts were measured using the *Folin*–*Ciocalteu* method in terms of gallic acid equivalent. The total polyphenols content of methanol extracts of *Leucaena leucocephala* waste was found to be 3.71 ± 0.1 mg of gallic acid/g of dried extract. This result was higher than the one reported from *Leucocephala* hybrid-Rendang [22].

### 3.2. Point of Zero Charge (pH_PZC_)

**Figure 1:**
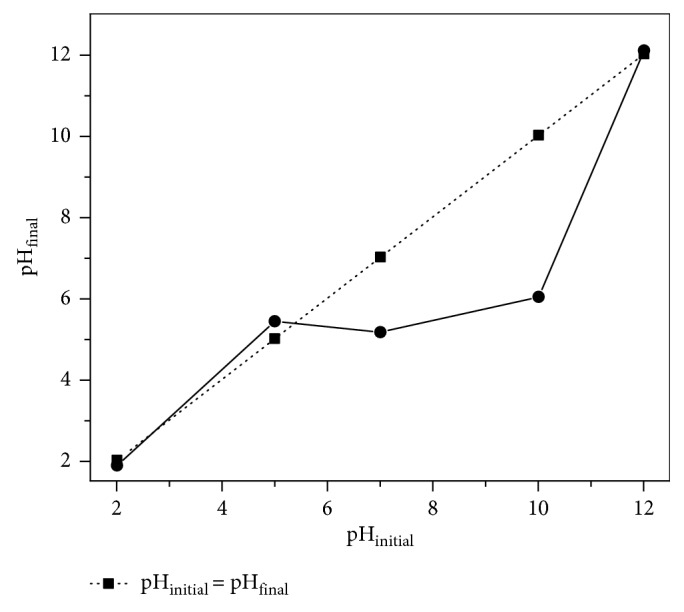
Point of zero charge (pHPZC) of LLEPs.

Figure 1 shows the point of zero charge results of LLEPs. The pH_PZC_ was found at pH = 5.10 ± 0.01; furthermore, a shift of the final pH (pH_f_) of the solution toward the slightly acidic region was observed, which could be attributed to the amphoteric character of the polyphenols surface that approximates the pH of a solution to their PZC value [27]. The effect of the amphoteric character of the surface can be observed in slight increase in the pH_f_ on the point of pH_i_ = 5. However, with respect to the surface charge of LLEPs, above the pH_PZC_ (pH > 5.1), the adsorbent surface is negatively charged, which could promote the attraction of cations, which is the case of our study. Therefore, it is plausible that a slight acidic range is favorable for metal retention onto LLEPs.

### 3.3. Mass Spectroscopy MALDI-TOF

**Figure 2:**
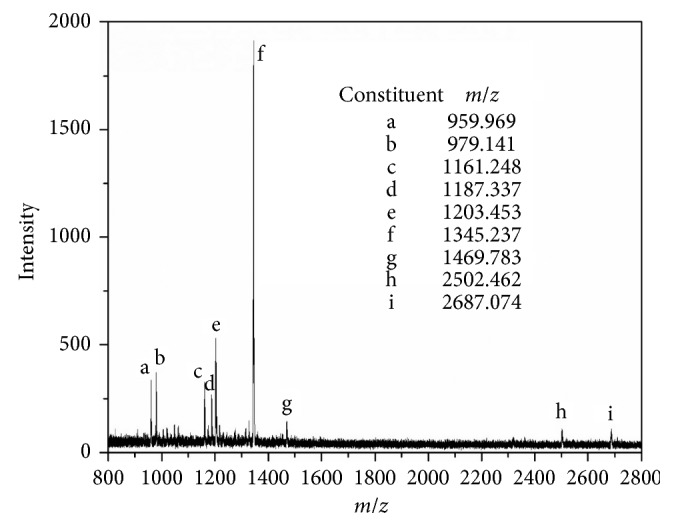
MALDI-TOF spectrum of the LLEPs.

To get more detailed information on the chemical composition and structure of natural compounds presented in LLEPs, MALDI-TOF mass spectroscopy analysis was performed. The flavonoidal constituents in the extracts of *Leucaena leucocephala* foliage have been identified earlier by mass spectrometry techniques as caffeic acid, isorhamnetin, chrysoeriol, isorhamnetin 3-O-galactoside, kaempferol-3-O-rutinoside, quercetin-3-O-rhamnoside, as well as luteolin-7-glucoside, among others [28–30]. Thus, in order to determine the different natural compounds in the extract of *Leucaena leucocephala* leaves studied in this work, we have analyzed the MALDI-TOF spectrum of a sample without using any cationizing agent (Figure 2). Considering that MALDI-TOF is not a quantitative method and the intensities of the signals do not necessarily reflect the fractions of the constituents in the sample, in this case, MALDI-TOF analysis is treated only as a confirmation of the presence or absence of natural products such as the aforementioned flavonoids in the tested sample.

**Figure 3:**
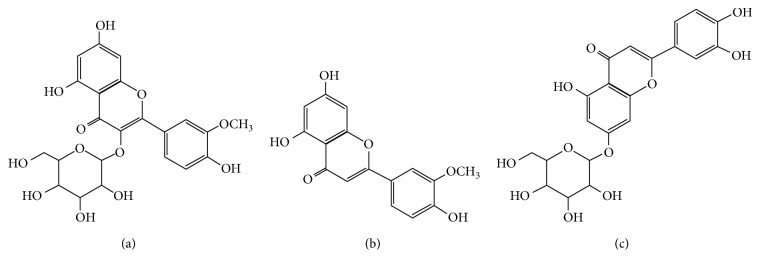
Base chemical structures of some natural compounds found in LLEPs: (a) isorhamnetin 3-O-galactoside, (b) chrysoeriol, and (c) luteolin-7-glucoside, respectively.

From Figure 2, it is observed that the signals of at least 9 populations of natural constituents are clearly dominating. For the dimers of isorhamnetin 3-O-galactoside, kaempferol-3-O-rutinoside, and chrysoeriol, the observed *m*/*z* values are 959.9, 1187.3, and 1203.4, respectively. The *m*/*z* signals for the trimers of luteolin-7-glucoside and quercetin-3-O-rhamnoside are both observed at 1345.2. Finally, for the tetramer of nonanoic acid, 9-(o-propylphenyl), methyl ester, the observed *m*/*z* value is 1161.2. The base chemical structures of some of these natural compounds are shown in Figure 3. It is worth noting that no data were found in the literature that would allow adequate identification of the natural compounds present in the sample with *m*/*z* values observed at 979.1, 1469.7, 2502.4, and 2687.0, respectively. Further research involving nuclear magnetic resonance spectroscopy (NMR) and the use of advanced sample purification techniques are necessary in order to clarify this issue.

### 3.4. ATR-FTIR Spectroscopy

**Figure 4:**
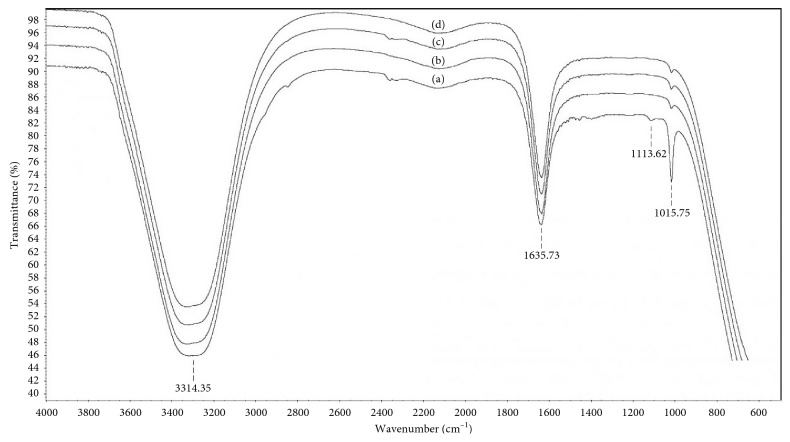
FT-IR spectra of LLEPs (a) before metal adsorption experiments (blank sample), (b) after Pb^2+^ adsorption experiment, (c) after Cd^2+^ adsorption experiment, and (d) after simultaneous Pb^2+^ and Cd^2+^ adsorption experiment, respectively.

ATR-FTIR spectroscopy was used to confirm the heavy metal adsorption carried out by LLEPs after 6 h of contact with cationic solutions of Pb^2+^, Cd^2+^, and the mixture of both metals, respectively. The comparative FTIR spectra before and after the different heavy metal adsorption experiments are shown in Figure 4. In order to better appreciate the differences in the absorption bands of the functional groups present in the chemical structure of the samples, the infrared spectra have been shifted by 3% from each other starting from the one corresponding to (d) (the sample taken after the simultaneous Pb^2+^ and Cd^2+^ adsorption experiment), followed by (c) (the sample taken after Cd^2+^ adsorption experiment), then (b) (the sample taken after Pb^2+^ adsorption experiment), and finally (a) (the sample taken before metal adsorption experiments).

Taking into account the base chemical structures elucidated by MALDI-TOF analysis for the LLEPs (Figure 3), one would expect for all of the samples the presence of characteristic absorption bands corresponding to the stretching vibrations of the O–H group around 3600 cm^−1^, the stretching vibrations of the =CH– (aromatic) group above 3000 cm^−1^, the stretching vibrations of the C=O (carbonyl) group around 1700 cm^−1^, and the stretching vibrations of the C–O (ether) group around 1100–1200 cm^−1^, among others. However, it is not possible to assign bands in the spectra of these samples unambiguously, as the absorbance bands are overlapping and poorly resolved. In this sense, since we have wet samples, the stretch signal H–O–H appears and overlaps the characteristic peak regarding the stretching vibrations of the O–H bond observed around 3314.35 cm^−1^ in Figure 4. Likewise, the representative flexion vibration band associated to H–O–H is also observed at 1635.73 cm^−1^.

It is worth noting that there are two well-resolved characteristic bands that appeared only in the blank sample (a) and clearly indicate that the heavy metal adsorptions have successfully taken place in the molecular architectures. For instance, the band displayed at 1113.62 cm^−1^ is attributed to the stretching vibrations of the C–O (ether) bond, whereas that shown at 1015.75 cm^−1^ is assigned to the stretching vibrations of the C–OH bond. As the metal adsorption has proceeded (b, c, and d), the absorption band due to the C–OH stretching decreases considerably, suggesting on the one hand that the LLEPs has effectively adsorbed the heavy metal ions and on the other hand that after 6 h of contact with the cationic solution active sites still remain to undergo metal adsorption. In contrast, the absorption band of aromatic C–O stretching completely disappears after deadsorption experiments pointing out that the ether moieties in the sorbent also participate effectively in the metal uptake. It is likely that steric hindrance plays an important role in the adsorption efficiency of the active sites described above. At this stage, FTIR analysis has been performed just in a qualitative fashion to corroborate the effectiveness of the sorbent to capture Pb^2+^ and Cd^2+^ from monocationic as well as polycationic solutions of both metals.

### 3.5. Kinetics

**Figure 5:**
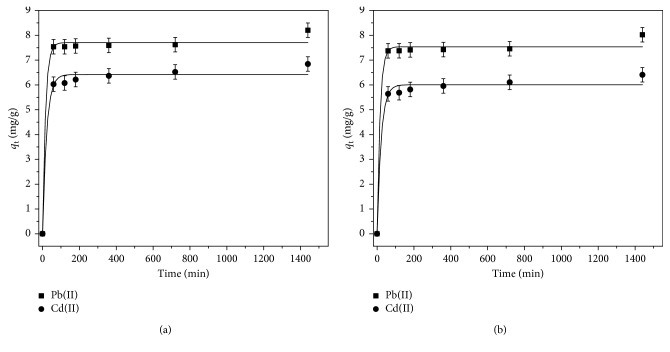
Effect of contact time on the adsorption percentage of Pb(II) and Cd(II) by LLEPs in (a) monometal and (b) bimetal systems (*C*_i_ = 100 mg/L; pH = 6).

The sorption equilibrium for Pb(II) and Cd(II) in mono- and bimetal system at pH = 6 by LLEPs was examined.  Figure 5 shows the plots of the sorption capacities of single and simultaneous Pb(II) and Cd(II), *q*(mg/g), as a function of time. It can be well seen that the adsorption increases sharply within the first 60 min while the equilibrium time (t_eq_) was reached within 120 min for both Pb(II) and Cd(II). The simultaneous adsorption in bimetal system showed a slight decrease in adsorption capacity due to the presence of a second metal whilst the equilibrium time did not changed. Generally, for both the cases, the adsorption process consisted of two main reaction stages, initial fast adsorption process, up to 65% of Pb(II) and 63% Cd(II) in mono- and bimetal system, followed by a slow continuous sorption reaction with 8–9% of metal. The first stage can be explained by the abundance of active binding sites that are gradually occupied by Pb(II) and Cd(II) as the contact time is increasing. In the second stage, the access to active sites becomes limited. This could be attributed to the possible metal ions repulsion from the LLEPs surface.

In order to describe the mechanism of sorption of Pb(II) and Cd(II) in mono- and bimetal systems onto LLEPs, the pseudo-first-order (Equation (2)), pseudo-second-order (Equation (3)), and Elovich (Equation (4)) models were used.

(2) $$\begin{array}{c} \ln\left(q_{\text{e}}-q_{\text{t}}\right)=\ln q_{\text{e}}-k_{1}t, \end{array}$$

(3) $$\begin{array}{c} \frac{t}{q_{\text{t}}}=\frac{1}{K_{2}q_{\text{e}}^{2}}+\frac{t}{q_{\text{e}}}, \end{array}$$

(4) $$\begin{array}{c} q_{\text{t}}=\;\;\frac{1}{\beta }\ln\left(\alpha \beta \right)+\;\;\frac{1}{\beta \;\;}\ln t, \end{array}$$

where *q*_t_ (mg·g^−1^) and *q*_e_ (mg·g^−1^) are the amounts of metal ions adsorbed at time *t* and equilibrium, respectively, *k*_1_ (min^−1^) and *k*_2_ (g.mg^−1^·min^−1^) are the equilibrium rate constants of pseudo-first-order and pseudo-second-order sorption process, respectively. *α* is the sorption rate (mg.g^−1^·min^−1^), and *β* is a desorption constant related to the extent of surface coverage and activation energy for chemisorption.  Table 1 shows the parameters of the kinetic models and correlation coefficients obtained from the lineal plots of ln(*q*_e_ − *q*_t_)  vs.  *t*  (Equation (2)), *t*/*q*_t_  vs.  *t* (Equation (3)), and *q*_t_  vs.  ln*t* (Equation (4)) of experimental data.

**Figure 6:**
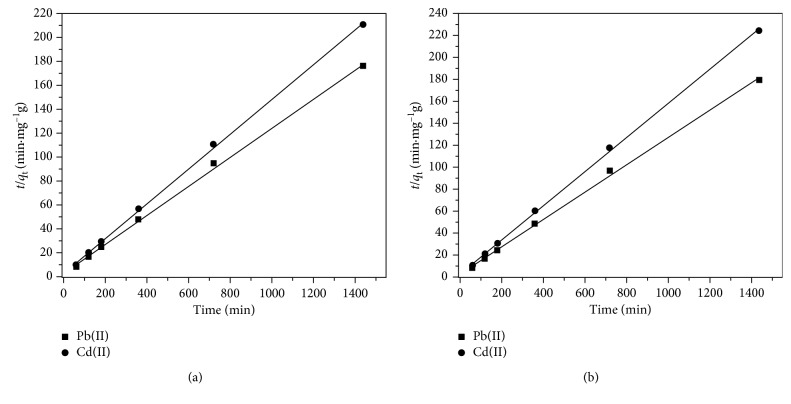
Pseudo-second-order kinetic plot for (a) single removal and (b) simultaneous removal of Pb(II) and Cd(II) sorption by LLEPs.

Figures 6(a) and 6(b) show the plot of *t*/*q*_t_ vs. *t*, for the single and simultaneous Pb^2+^ and Cd^2+^, respectively. As seen from Figure 6, the experimental data well adjusts to the pseudo-second-order kinetic model (R^2^ ≥ 0.999). Moreover, it can be observed that *q*_m_ values calculated from Equation (3) are similar with the experimental *q*_m_ values (Table 1). This indicates that the sorption kinetics of Pb(II) and Cd(II) show better agreement for the pseudo-second-order model. Similar results are reported in the literature, where mentioned that in most cases of biosorption, pseudo-second-order equation fit better for the whole range of contact times than the pseudo-first-order [16].

### 3.6. Effect of Polyphenols Dosage

**Figure 7:**
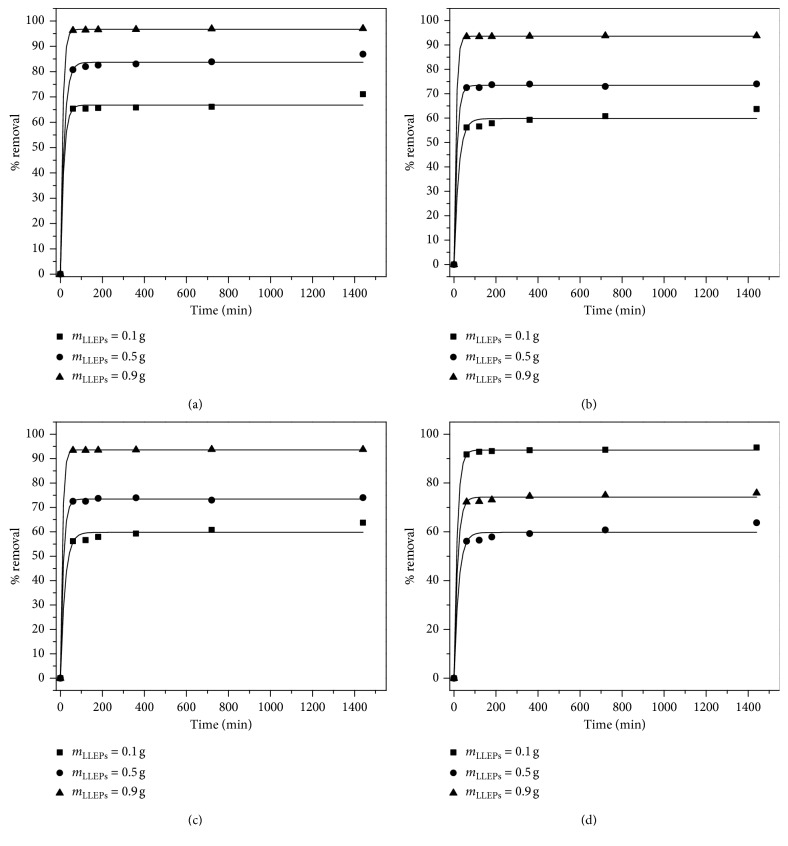
Effect of LLEPs dosage on the removal percentage of Pb(II) and Cd(II) in (a, b) monometal and (c, d) bimetal systems (*C*_i_ = 100 mg/L; pH = 6).

The study of the effect of LLEPs dosage is essential as the adsorbent dosage showed to be an important parameter that influences the metal uptake from the solution.  Figure 7 shows the initial mass introduced of LLEPs as a function of time; the results showed that the percentage removal of metal ions increases with increasing the amount of LLEPs 0.1 g to 0.9 g in mono- and bimetal systems; This could be attributed to the availability of active binding sites.

As the Figures 7(a) and 7(b) show, 0.9 g of LLEPs was enough to remove over 98% of Pb(II) and Cd(II) (*C*_i_ = 100 mg/L) individually, whilst 0.1 g of LLEPs uptake was limited in 65% for these metals. Similarly, in bimetal system, LLEPs showed the same behavior but with a slight decrease in removal uptake, where 55% of Pb(II) and Cd(II) was removed by 0.1 g, while 0.9 g of LLEPs was able to remove 95% of both metals. In general, the removal of Pb(II) was faster than Cd(II) ions as it will be discussed in the next section.

### 3.7. Isotherms

**Figure 8:**
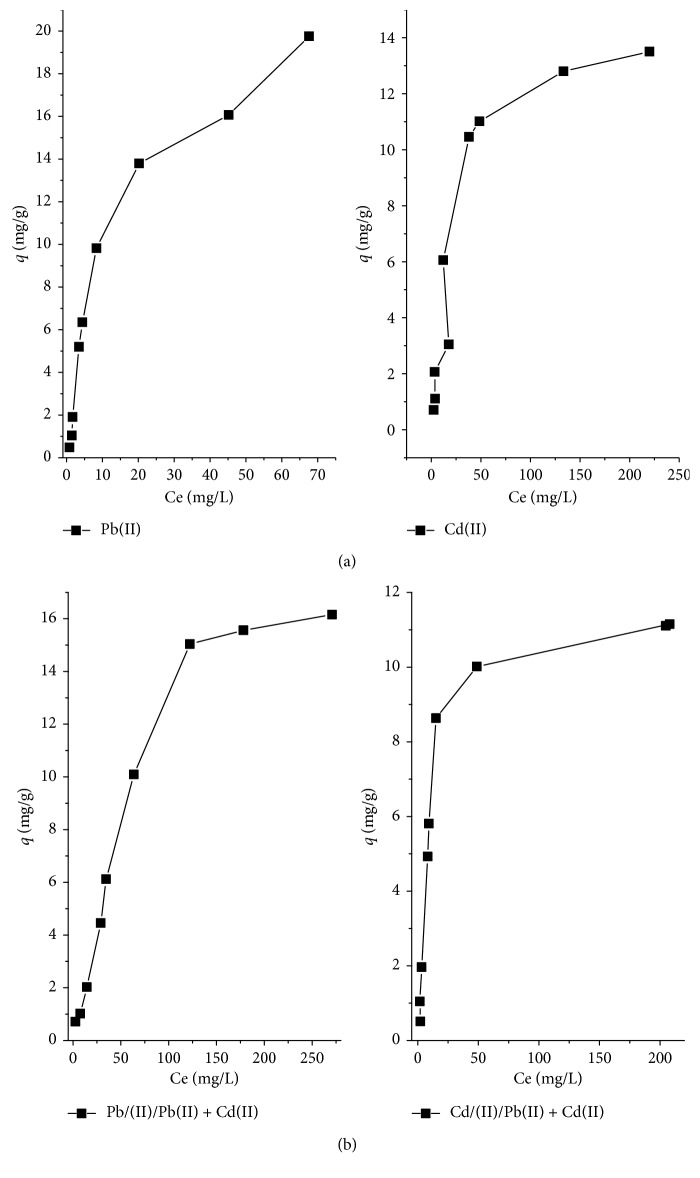
Pb(II) and Cd(II) sorption isotherms by LLEPs in (a) monocomponent and (b) bicomponent systems.

Figure 8 presents the isotherms of Pb(II) and Cd(II) in mono- and bimetal system at pH = 6 by LLEPs. The metal removal capacity as a function of metal concentration at the equilibrium state was analyzed using Langmuir and Freundlich models. The Langmuir isotherm describes monolayer adsorption on the surface of an adsorbent with a finite number of identical adsorption sites and no interaction between sites. This model is represented as follows:

(5) $$\begin{array}{c} \frac{C_{e}}{q_{e}}=\frac{1}{q_{\text{m}}K_{\text{L}}}+\frac{C_{e}}{q_{\text{m}}}\;\;, \end{array}$$

where *q*_m_ (mg·g^−1^) is the maximum adsorption capacity of the monolayer and *K*_L_ (L·mg^−1^) is the Langmuir adsorption equilibrium constant related to the energy of adsorption. *q*_m_ and *K*_L_ were determined from the slope and intercept of the plots of *C*_e_/*q*_e_ versus *C*_e_.

The Freundlich isotherm is an empirical equation used to describe heterogeneous systems, and it is expressed by the following linear equation:

(6) $$\begin{array}{c} \ln q_{\text{e}}=\ln K_{\text{F}}+\frac{1}{n}\ln C_{\text{e}}, \end{array}$$

where *K*_F_ (mg·g^−1^) (L·mg^−1^)^1/*n*^ is the Freundlich isotherm constant and *n* is the adsorption intensity. A plot of log *q*_e_ versus log *C*_e_ gives a straight line of slope 1/*n* and intercepts log *K*_F_.

In the present work, the experimental data were fitted to the Langmuir model for describing the obtained isotherms to propose the sorption mechanism involved with the highest determination coefficient values, *R*^2^ (0.980, 0.998, 0.996, and 0.968) when compared with the Freundlich isotherm. However, from Table 2, it is clear that the maximum adsorption capacity of Pb(II) individually by LLEPs (*q*_mPb_ = 25.510 mg/g) is higher than that observed in the presence of the second metal (*q*_mPb/Pb+Cd_ = 21.552), whereas the maximum Cd(II) adsorption capacity showed the reverse results when it was higher in bimetal system (*q*_mCd/Cd+Pb_ = 16.807) than monometal system (*q*_mCd_ = 14.792). The constant related to the affinity of the binding site (*K*_L_) is lower for competent solution metals compared to the one with individual metal, which is in agreement with the abundant binding sites in the latter.

It is generally well known that the specific adsorption of metals on different adsorption surfaces is dependent on their hydration energies; however, higher hydration energy of cations implies higher hydration radii. From the above results, the adsorption capacity toward Pb(II) was obviously higher as compared to Cd(II) due to big coordination sphere offered in its case since it has an energy of hydration higher (Δ*H*_*h,Pb(II)*_ = −1485 kJ/mol) than Cd(II) (Δ*H*_*h,Cd(II)*_ = −1809 kJ/mol), thus, an ionic radius (1.19 Å) higher than Cd (0.95 Å) [31]. Hence, Pb(II) ions can bind better with polyphenols groups. Moreover, the electronegativity also has an important role in the preference of biosorbents for metals; however, as the metal is more electronegative, it will be rapidly attracted to the negative surface of a biosorbent, in our case, to the negative-charged surface of LLEPs at the studied pH as confirmed by PZC measure. Therefore, as the lead cation Pb(II) provides the higher electronegativity (2.33) than the cadmium Cd(II) with value of 1.69, the Pb^2+^ ions were rapidly diffused to the LLEPs surface negatively charged.

## 4. Plausible Mechanism

**Figure 9:**
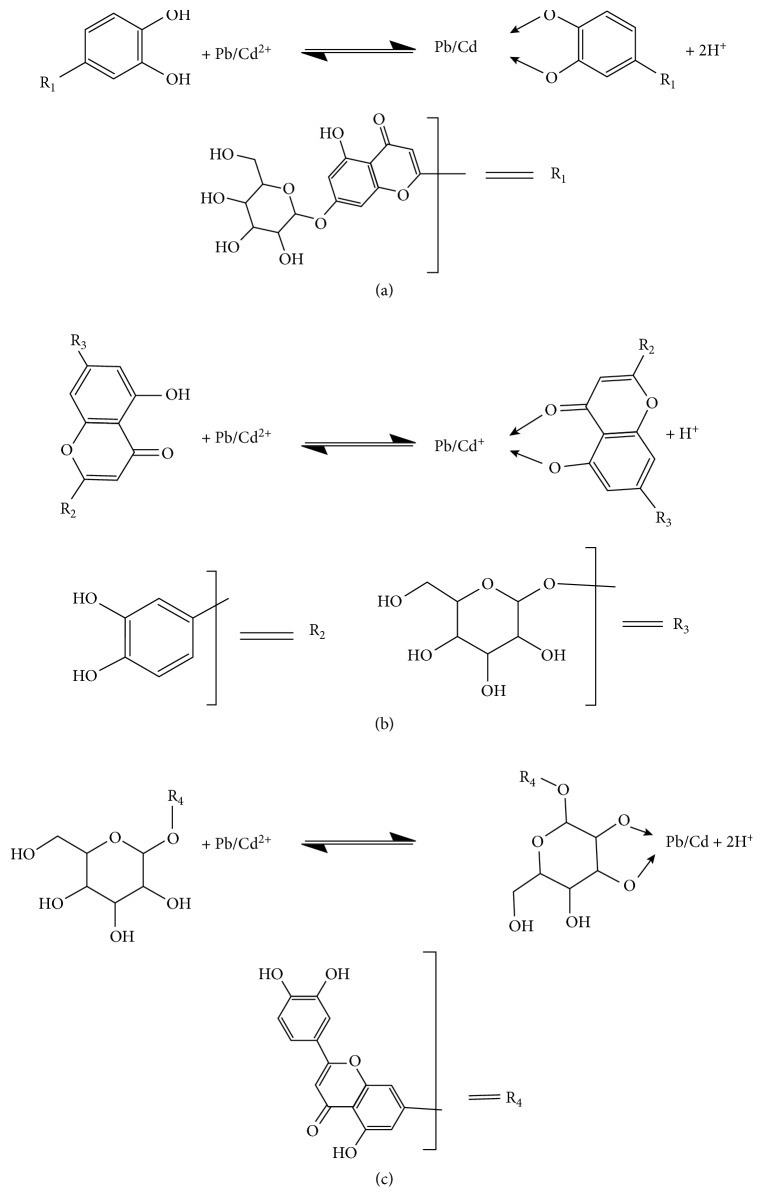
Inferred mechanism for M = Pb(II) or Cd(II) adsorption as well as complex formation with (a) catechol, (b) hydroxy and carbonyl, and (c) hydroxyls groups presents in LLEPs.

Polyphenols have been widely reported as metal scavengers such as flavonoids, stilbenes, and lignans compounds [32–34]. In our case, three potential metal-binding sites are identified in flavonoidal compounds of LLEPs (Figure 9). It is well known that the deprotonated phenolic is a particularly good ligand for metal cations, once the oxygen center is generated possessing a high charge density, called *Hard ligand* (Figure 9(a)). On the contrary, the presence of a C=C double bond conjugated with a 3-oxo group is also of great importance for partial delocalization that could also bind heavy metal cations (Figure 9(b)) [35]. Besides, the hydroxyl groups presented the molecules R-C_6_O_6_H_9_ is probably involved in the biosorption process of heavy metals as suggested in the Figure 9(c). However, the major contribution to metal chelation is due to the catechol moiety (Figure 9(a)) owing to the presence of two hydroxyl groups in *ortho-*positions provides a strong metal-chelating character [36].

Therefore, since lead (Pb^2+^) and cadmium (Cd^2+^) were effectively sorbed onto the LLEPs, they should combine with hydroxyl ions (–OH) and pyrone oxygen of flavonoids compounds as it is exemplified in luteolin-7-glucoside. In general, the binding of metal ions on polyphenolic groups as well as alcoholic groups was considered to take place by the ion-exchange mechanism leading to the formation of a 5-membered chelate complex. A similar phenomenon for Cs(VI)-catechol complex has also been reported [37].

## 5. Conclusions

From this work, it can be concluded that L. *leucocephala*-extracted polyphenols are an effective low-cost biosorbent of Pb(II) and Cd(II) in monosystem and binary system.

Successful extraction of polyphenols from *Leucaena leucocephala* plant was identified by MALDI-TOF mass spectroscopy, with high rate compared to reported results of this plant.

ATR-FTIR spectroscopy clearly indicated that the heavy metal adsorptions have successfully taken place in the molecular architectures.

Nearly 100% removal of lead and cadmium was possible at a neutral pH value by 0.9 g of LLEPs, while 95% removal of the mixture Pb-Cd was possible at a pH value of 5 under the batch test conditions.

The pseudo-second-order model best describes the sorption behavior of Pb(II), Cd(II), and their mixture, and the *k*_2_ values depend on the adsorbent mass. This implied that the chemisorption process might be the mechanism of the solute ion-LLEPs interaction in this research work.

The experimental data of both metals Pb(II) and Cd(II) sorption isotherms well fit the Langmuir model. The *K*_L_ parameter is lower for competent solution metals compared to the one with individual metal. The maximum adsorption capacity (*q*_m_) of Pb(II) ions was obtained as 25.51 and 21.55 mg/g in mono- and bimetal solutions.

## Figures and Tables

**Table 1:** Kinetics parameter of Pb(II) and Cd(II) removal in both mono- and bicomponent systems by different volumes of LLEPs.

*m*_LLEPs_ (g)	Metallic ions	Pseudo-second-order				Pseudo-first-order			Elovich model		
		*q*_e_ (cal)	*q*_e_ (exp)	*k*_2_	*r* ^2^	*q*_e_ (cal)	*k*_1_	*r* ^2^	*α*	*β*	*r* ^2^
0.1	Single removal										
	Pb^2+^	8.203	8.199	0.007	0.999	1.497	0.004	0.814	0.008	0.288	0.584
	Cd^2+^	6.887	6.844	0.007	0.999	1.105	0.002	0.957	0.003	0.267	0.954
	Simultaneous removal										
	Pb^2+^	8.025	8.021	0.006	0.999	1.476	0.004	0.814	0.009	0.282	0.584
	Cd^2+^	6.447	6.404	0.007	0.999	1.029	0.002	0.959	0.043	0.250	0.954

0.5	Single removal										
	Pb^2+^	2.008	2.003	0.042	0.999	0.192	0.002	0.908	4.329	0.043	0.906
	Cd^2+^	1.588	1.589	0.285	0.999	0.022	0.002	0.461	13.70	0.016	0.686
	Simultaneous removal										
	Pb^2+^	1.872	1.869	0.204	0.999	0.044	0.002	0.995	12.83	0.013	0.947
	Cd^2+^	1.530	1.525	0.101	0.999	0.088	0.002	0.979	10.06	0.026	0.960

0.9	Single removal										
	Pb^2+^	1.243	1.242	0.798	0.999	0.011	0.002	0.995	94.10	0.003	0.996
	Cd^2+^	1.118	1.117	1.427	0.999	0.005	0.002	0.961	184.6	0.002	0.967
	Simultaneous removal										
	Pb^2+^	1.201	1.200	0.748	0.999	0.012	0.002	0.946	54.59	0.006	0.795
	Cd^2+^	1.055	1.054	0.278	0.999	0.033	0.002	0.931	39.88	0.009	0.927

**Table 2:** Langmuir isotherm constants for single and simultaneous Pb(II) and Cd(II) removal by LLEPs.

Metallic ions	Langmuir isotherm		
	*q*_m_ (mg/g)	*K*_L_ (dm^3^/mg)	*r* ^2^
*Single removal*			
Pb(II)	25.510	6.112	0.980
Cd(II)	14.792	55.793	0.998

*Simultaneous removal*			
Pb(II)	21.552	2.269	0.996
Cd/II	16.807	12.549	0.968

## Data Availability

The authors declare that they have no inconvenience to share the data generated on this study.
